# “A Word with the Doctors,” from the “Western Lancet”

**Published:** 1856-01

**Authors:** 


The January number of the Western Lancet contains an article headed "A Word with the Doctors" which we hope they may all read. It is by Dr. W. A. PEASE, of Dayton, Ohio.

In speaking of the present and former condition of Dentistry, Dr. Pease remarks as follows.

"A few years since, dentists and physicians occupied nearly the same ground in the treatment of the teeth. There were but few instruments, and they were illy adapted to our wants; and there was no pharmacopia belonging to or generally used by the profession. The dentist was synonymous with the manufacture of artificial dentures; he did little more than extract aching teeth, or, if he attempted to arrest disease by plugging, it was often so imperfectly done as to be but little more than of temporary value—deferring the loss of the teeth but a few years. When caries had penetrated to the nerve, the tooth was beyond his resources. If we consider the amount of disease that occurs low down on the approximal surface of the teeth near the gum, where it is masked or difficult to discover, even when the health of the teeth is a matter of care, and that, in those places, there is but a comparatively thin portion of bone for disease to traverse before reaching the pulp—while the accelerating influence of decomposing food on caries is here the greatest, the great loss of the teeth will be apparent, especially of the molars and bicuspids, and the necessary amount of mechanical dentistry, where so large a proportion of the teeth are liable to so common and serious a disease, and that disease is incurable. Dental statistics, so far as they have been tabulated, show that the great loss of the teeth is in early life, before the system becomes consolidated, or soon thereafter. It is not uncommon, for the first permanent molar, which cuts at six years of age, to be so diseased as to be beyond remedy at nine; and the other permanent teeth often as rapidly follow. Thus, in some 1,300 permanent bicuspid and molar teeth I extracted consecutively, 32 per cent. were extracted from the mouths of persons under twenty-five years of age, and 31 per cent. under thirty, and a considerable per cent. of the remainder was for persons under twenty years of age. If, in addition to this, the large number of teeth plugged for persons under thirty years of age is taken into consideration, the frightful amount of disease in early life will be apparent."

The following remarks in relation to Mechanical Dentistry are very appropriate.

"The prevalent opinion among physicians, which has spread from them to the community is, that dentistry is purely mechanical, that a man, who can make a set of teeth, *is a dentist*, and that in *that* consists the chief skill of dental practice. Physicians by their action as a body, and individually, are responsible for much of the public estimation of dental ability, and the value attached to many operations; viz.: the weight of the profession has always been in favor of extracting aching teeth. Caries has penetrated to the nerve of a tooth without burrowing much, or undermining the enamel, the cavity is merely a tube, the strength of the tooth is not much impaired, but the tooth aches; without the intervention of dentists the rule would be to extract the tooth; yet by destroying and removing the nerve, the tooth may be made valuable for a period of time if not for life. After the tooth has been plugged there is perhaps tenderness at the apex of the fang, possibly absolute pain. The physician cries ulceration! ulceration! foreign substance! dead tooth! it must come out! and so impresses the patient, that it is difficult to disabuse him, and make him believe the difficulty is only temporary, with little likelihood there will be more than a passing irritation, arising from the efforts of the overtaxed absorbents to remove some remaining particles of the dead nerve. Instead of helping nature by stimulating the action of the absorbents and keeping down irritation, he adds fuel to the fire by hot preparations, yet often after a deal of grumbling the tooth gets well in spite of him. Thus the physician creates a public opinion unfavorable to treatment of aching teeth—to conservative dentistry in general; and then because that public opinion demands mechanism, he calls us mechanics, and mechanical dentistry; thus fortified, like Jonah's gourd, swallows up the conservative. It is not strange that mechanical dentists, conscious of their own fate, should, like monarchs, seek to perpetuate their succession and avail themselves of the proclivities of the physician in their favor. Tooth-ache with them is an ugly customer, and plugs do not pay; so they adopt the maxim, that extracted teeth, like dead men, tell no tales; while inserting teeth is a palpable, attractive, walking advertisement, and they now have their sign board in the shape of artificial dentures in the mouths of half of the women in fashionable society. In contrast with them, here and there, scattered through the land, are a few quiet individuals, patiently studying the physiology and pathology of the mouth; carefully making experiments and noting the results. Emboldened little and little by success, they have progressed till they feel competent to preserve a large class of decayed teeth for practical purposes. But, unfortunately, the way has been barricaded against them, the community are prejudiced against large plugs, and the weight of medical testimony is against them. The mechanical dentist fortifying himself, inserts none, and for the small plugs he does insert, he charges so low, that no one can make a business of plugging teeth, and thus acquire the skill resulting from constant practice and concentrated attention to it, to say nothing of the more difficult classes of teeth, and live. With few exceptions good pluggers are found only in cities, and then often but a few individuals, who are obliged to eke out a living, or look for their greatest profit to their workmen in the laboratory. I have frequently asked the most accomplished pluggers how many plugs they could insert in a day; they have generally answered, about 8, (a mechanic will insert 20.) Now the ruling price throughout the northern States is for gold $1 per plug, for tin 50 cents. Tin is often inserted in large and difficult cavities on account of economy both to the patient and dentist, and consequently demands more labor, making the average receipts of the honest, skilful dentist respectively 4 and 8 dollars, according to the material. The greater cost of the gold often makes tin the most profitable, so that practically, the man who brings to the profession an education worthy of it, and of a professional man, must furnish an office, provide an expensive set of instruments, and, if he confines himself to legitimate practice, subsist on hod carriers wages; unless he is fortunate in having liberal friends.

